# Randomized study to prove the quality of human ovarian tissue cryopreservation by xenotransplantation into mice

**DOI:** 10.1186/s13048-019-0521-5

**Published:** 2019-05-21

**Authors:** Xiangyan Ruan, Yamei Cui, Juan Du, Jing Jin, Muqing Gu, Suwen Chen, Alfred O. Mueck

**Affiliations:** 1 Department of Gynecological Endocrinology, Beijing Obstetrics and Gynecology Hospital, Capital Medical University, No. 251, Yaojiayuan Road, Chaoyang District, Beijing, 100026 China; 2 Department of Women’s Health, University Women’s Hospital and Research Center for Women’s Health, University of Tuebingen, Tuebingen, Germany; 3 Department of Family Planning, Beijing Obstetrics and Gynecology Hospital, Capital Medical University, Beijing, China

**Keywords:** Human cryopreserved ovarian tissue quality, Mouse model, Follicular growth rate, Follicular survival rate

## Abstract

**Purpose:**

To study the quality of our human ovarian tissue cryopreservation technique as performed in the first official "International Fertility Protection Centre" in China in patients with certain cancer types using a mouse model, and to find the best site for tissue transplantation in the mouse.

**Methods:**

Thirty-six BALB/C female nude mice were randomly divided into 3 groups, group 1: control group; group 2: ovariectomized group; group 3: ovarian tissue transplantation group. Seventy-two pieces obtained from six ovarian tissue samples from each of three cancer patients were transplanted into the ovarian bursa cavity (OBC), the subcutaneous thigh (TS) and the subcutaneous neck (NS) and removed after 1.5 and 2.5 months, respectively. Follicular growth rate (FGR), total follicle surviving rate (TFSR), tissue recovery rate (TRR), antral follicles (AF), follicle stimulating hormone (FSH), estradiol (E2) and anti-Mullerian hormone (AMH) levels were measured.

**Results:**

No significant differences in FGR, OBC, NS (*p* > 0.05); TFSR was 100% in OBC, NS and TS. No significant differences in TRR (*p* > 0.05); AF were found only in OBC; TFSR was 100% after transplantation; significantly higher FGR in the 2.5 months compared to the 1.5 months-group (*p*  < 0.05). AMH- and E2-level in group 1 and 3 were significantly higher than in group 2 (*p* < 0.05); in contrast, FSH was significantly lower.

**Conclusions:**

After transplantation in the mice, the thawed ovarian tissue survived and follicles developed. The ovarian fossa site was the best site for transplantation. Our animal experiments can verify that our human ovarian tissue cryopreservation technique can preserve the quality of ovarian tissue. This is the essential precondition for successful re-transplantation into the patients after performing chemo/radiotherapy to protect ovarian function and fertility.

## Introduction

Several factors such as advanced diagnostic tools, early cancer detection, and aggressive cancer therapies have increased the current 5-year survival rate for adults and children (80%) [1, 2]. However, chemotherapy and/or radiotherapy very often lead to partial or complete impairment of the ovaries, severely reducing or eliminating a woman’s fertility [3–5]. Before oncological treatment, a promising option for preserving fertility is the cryopreservation of ovarian tissue in women and prepubertal girls with certain forms of cancer [6–10]. It is a clear option to store a high number of primordial and primary follicles and can be performed quickly at any time during the menstrual cycle without delaying oncological treatment. These follicles can grow and develop after grafting of frozen-thawed ovarian fragments. This technique for preserving the fertility of cancer patients has become clinical routine in some European countries and American states. It is expected that in the near future more and more cancer patients are likely to request reimplantation of cryopreserved ovarian tissue [11–13]. Over 130 live births have been reported worldwide after auto-transplantation of frozen-thawed ovarian cortex [6, 14].

Xenografting of human ovarian tissue to mice has already been proven to be an effective model to study ovarian function and follicle development in vivo [1]. For this reason, the aim of our study was to use xenotransplantation into mice to evaluate the quality and functionality of cryopreserved human ovarian tissue from cancer patients. The main aim was to test the quality of the cryopreserved tissue in the mouse model after thawing, the second aim was to find the best site for transplantation into the mouse for daily routine, thereby verifying the quality of the cryopreservation and thawing technique in our laboratory. The most appropriate site for an adequate transplantation has not yet been clearly determined. This site may also not be the best place for re-transplantation in women, which is another open question and is not the aim of our study. For practical reasons and because of the good blood supply, we chose the ovarian bursa cavity (OBC), the subcutaneous thigh (TS) and the subcutaneous neck (NS) as transplantation sites in our mouse model for testing the quality of tissues obtained from cancer patients within a prospective randomized three-arm study. Since the duration of xenotransplantation, which is needed to assure good quality control, has also varied in other studies, our aim was to also investigate this question. According to the literature, this time ranges between 1 week and 28 weeks [15], and we therefore decided to choose a time between this range and divided the mice into two groups with 1.5 and 2.5 months transplantation, respectively.

In our hospital, the first official “International Fertility Protection Centre” in China which specialises in using ovarian tissue cryopreservation has been established, and cooperates especially with experts on this technique in Germany (FertiProtekt® network) [16]. We recently published the first case report of a clinically successful re-transplantation [17]. This study is the first in China to test the quality of tissue obtained from cancer patients within an animal model after cryopreservation to achieve clinically successful re-transplantations.

## Materials and methods

### Human ovarian tissue

Human ovarian tissue samples were collected from three patients diagnosed with cervical cancer after obtaining written informed consent. The patients did not have chemotherapy prior to laparoscopic surgery. The mean age of the patients was 31.00 ± 1.73 years (29–32 years) at the time of ovarian tissue cryopreservation. Prior to laparoscopic retrieval of ovarian tissue from the patient**,** FSH (5.36 ± 1.07 IU/L) and E_2_ (76.83 ± 27.68 pg/ml) were determined using commercially available ELISA kit (FanKe Biology Technology Co., Shanghai, China).

### Ethical approval

Use of human ovarian tissues from three cancer patients for this study was approved by the Ethics Committee of the Capital Medical University, China. The Ethics Committee agreed that slices of the patients’ tissues can be used for patient-related research studies. The Ethics Committee confirmed that for testing the biological quality of tissue, in vivo experiments, i.e. animal experiments, are needed.

All procedures performed in studies involving human participants (in our study getting the tissue from three cancer patients) were in accordance with the ethical standards of the institutional and/or national research committee and with the 1964 Helsinki declaration and its later amendments or comparable ethical standards.

All procedures performed in studies involving animals were in accordance with the guidelines approved by the Animal Experiments and Experimental Animal Welfare Committee of Capital Medical University, China (AEEI-2015-017). These guidelines comply with the ARRIVE guidelines, in accordance with the National Institute of Health guide for the care and use of laboratory animals (NIH Publications No.8023, 1978).

### Animals

Thirty-six 8 week old female BALB/c nude mice were obtained from Capital Medical University, China. The BALB/c nude mice lack a thymus, are unable to produce T-cells and are therefore immunodeficient. Four mice were housed per cage (3 cages in each group) with high efficiency particulate air filters with a temperature and light (12-h light, 12-h darkness) controlled environment in individually ventilated cages (IVC). Animals had free access to water and food. Animals were allowed acclimatise for 1 week.

### Collection of human ovarian tissue

Human ovarian tissue samples were obtained laparoscopically and immediately transported on ice to the human ovary tissue cryopreservation bank laboratory, which was recently established in the Beijing Obstetrics and Gynaecology Hospital, Capital Medical University. The ovarian tissue samples were tested using our routine microscopic and immunohistological evaluations which showed that the ovarian tissue samples did not contain metastases.

### Human ovarian tissue freezing and thawing and preparation

Ovarian tissue fragments and strips (3 × 3 mm) were prepared under sterile conditions. The freezing procedure was performed using a slow-cooling protocol with a freezing system developed at the University of Bonn, Germany [18]. Under aseptic conditions, cryogenic vials were thawed at room temperature for 30 s then immersed in a 37 °C water bath. Afterwards, the tissue samples were transferred from the vials to tissue culture dishes and washed stepwise in medium containing DPBS, serum substitute supplements to remove cryoprotectant. Thawed cortical fragments were then transported to the animal facility of the Capital Medical University where we performed the animal experiments.

### Xenotransplantation procedure

Twelve mice without any surgeries were grouped into the control group. Twelve ovariectomized mice were grouped into an ovariectomized group as a second control group. Twelve ovariectomized and human ovary tissue xenotransplanted mice were grouped into the transplantation group. Twenty-four nude mice were anaesthetized with 40 mg/kg body weight chloral hydrate. The surgery was performed on a warming plate in a sterile environment. A dorsomedial skin incision was performed for the bilateral ovariectomy. After ovariectomy, six 3-mm frozen-thawed human ovarian tissue pieces (tissue strips) were placed differently into subcutaneous neck-pouch, ovarian bursa cavity and subcutaneous thigh sites. Six cortical tissue slices obtained from each of the three patients were divided into 24 tissue strips for transplantation into the 12 mice (each received 6 strips for the 3 different sites), giving a total of 72 tissue strips. The grafts were fixed with absorbable sutures. After grafting, 12 nude mice were randomized to be observed either for 1.5 months or 2.5 months.

At the end of each observation period, the animals were sacrificed. The recovered grafts were fixed in 4% formalin(Kang Naixin Bio Medical Technology Co, China). The uteri of the 3 groups were removed, fixed in 4% formalin and embedded in paraffin(Xinji Jingshan Petrochemical Plant, China) for histological assessment.

### Follicle counting

Histological analysis was performed after the grafts were fixed in 4% formalin and embedded in paraffin. The strips were dehydrated, embedded in paraffin and cut into serial sections. Haematoxylin and Eosin (H&E, Beyotime, China) were used to stain each section for follicle classification and counting [19]. Follicular development was assessed with the × 40 objective of a light microscope as described by Myers et al. [20]. Follicles were counted in the whole fragment and classified according to stage into primordial, intermediate, primary, secondary or antral follicles. Because only five oocyte nuclei were seen, we mainly counted the follicles. Definition of the follicles was as follows: primordial follicle - one layer of flattened granulosa cells, primary follicle - one layer of cuboidal granulosa cells, secondary follicle - two or more layers of granulosa cells. An antral follicle was defined by antrum formation. Intermediate follicles had both flattened and cuboidal granulosa cells. Only follicles with a visible nucleus in the oocyte were counted [21].

Observation indexes used in this study were as follows:

$$ {\displaystyle \begin{array}{c}\mathrm{TFSR}\ \left(\mathrm{total}\ \mathrm{follicle}\ \mathrm{surviving}\ \mathrm{rate}\right)=\\ {}\left(\mathrm{primordial}\ \mathrm{follicle}+\mathrm{primary}\ \mathrm{follicle}+\mathrm{secondary}\ \mathrm{follicle}+\mathrm{antral}\ \mathrm{follicle}\right)/\\ {}\left(\mathrm{primordial}\ \mathrm{follicle}+\mathrm{primary}\ \mathrm{follicle}+\mathrm{secondary}\ \mathrm{follicle}+\mathrm{antral}\ \mathrm{follicle}+\mathrm{atretic}\ \mathrm{follicle}\right)\end{array}} $$

$$ {\displaystyle \begin{array}{c}\mathrm{FGR}\ \left(\mathrm{follicle}\ \mathrm{growth}\ \mathrm{rate}\right)=\left(\mathrm{primary}\ \mathrm{follicle}+\mathrm{secondary}\ \mathrm{follicle}+\mathrm{antral}\ \mathrm{follicle}\right)/\\ {}\left(\mathrm{primordial}\ \mathrm{follicle}+\mathrm{primary}\ \mathrm{follicle}+\mathrm{secondary}\ \mathrm{follicle}+\mathrm{antral}\ \mathrm{follicle}\right)\end{array}} $$

$$ \mathrm{TRR}\ \left(\mathrm{tissue}\ \mathrm{recovery}\ \mathrm{rate}\right)=\mathrm{the}\ \mathrm{number}\ \mathrm{of}\ \mathrm{recovered}\ \mathrm{ovarian}\ \mathrm{tissue}/\mathrm{the}\ \mathrm{number}\ \mathrm{of}\ \mathrm{xenotransplanted}\ \mathrm{ovarian}\ \mathrm{tissue} $$

### Blood test

Before the grafts were recovered, blood samples were collected by orbital sinus puncture at the end of the observational period and at the end of the study. The blood was centrifuged by 3000 r/min to obtain the serum. Then the serum was refrigerated at − 80 °C. The blood follicle stimulating hormone (FSH), estradiol (E2) and anti-Mullerian hormone (AMH) levels were measured by electrochemical luminescence and analysed by variance analysis (FanKe Biology Technology Co., Shanghai, China).

### Statistics

Statistical analysis was performed using the SPSS software package (version 19; SPSS, Inc., China). All data is represented as mean ± SD. The follicle counts were analysed with the chi-square test. The level of FSH, E2 and AMH were analysed with variance analysis. Statistical significance was confirmed by *p* values < 0.05.

## Results

**Fig. 1:**
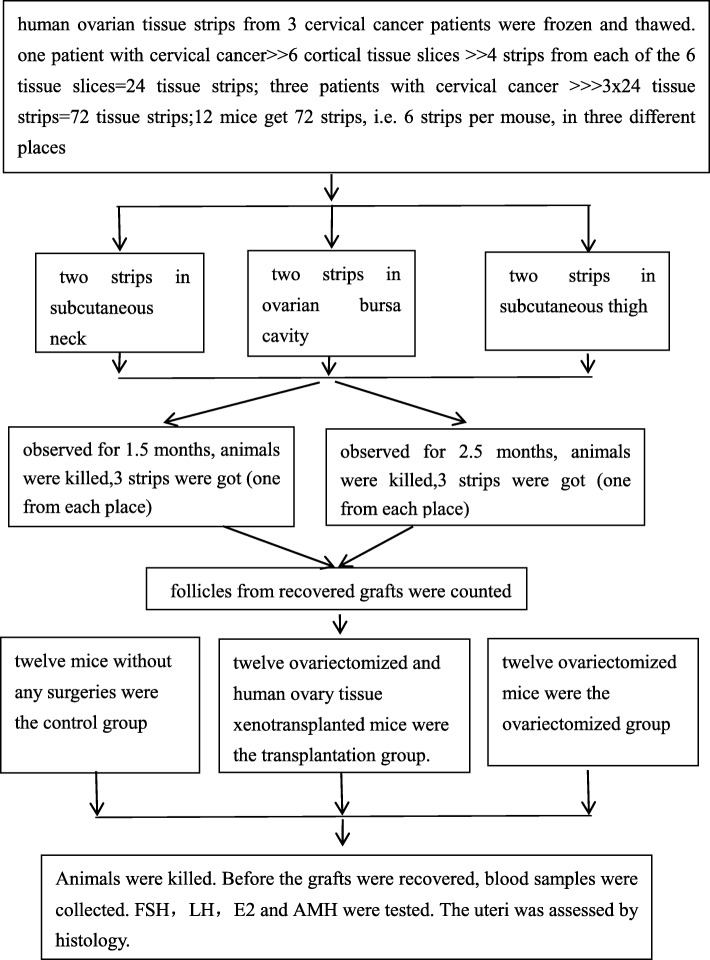
Diagram of the experimental design and allocation of the mice to three groups

Figure 1 shows the diagram of the experimental design and the allocation of the mice to the three groups, control group, transplantation group and ovariectomized group.

### Histology of ovarian tissue after transplantation in each group

**Fig. 2:**
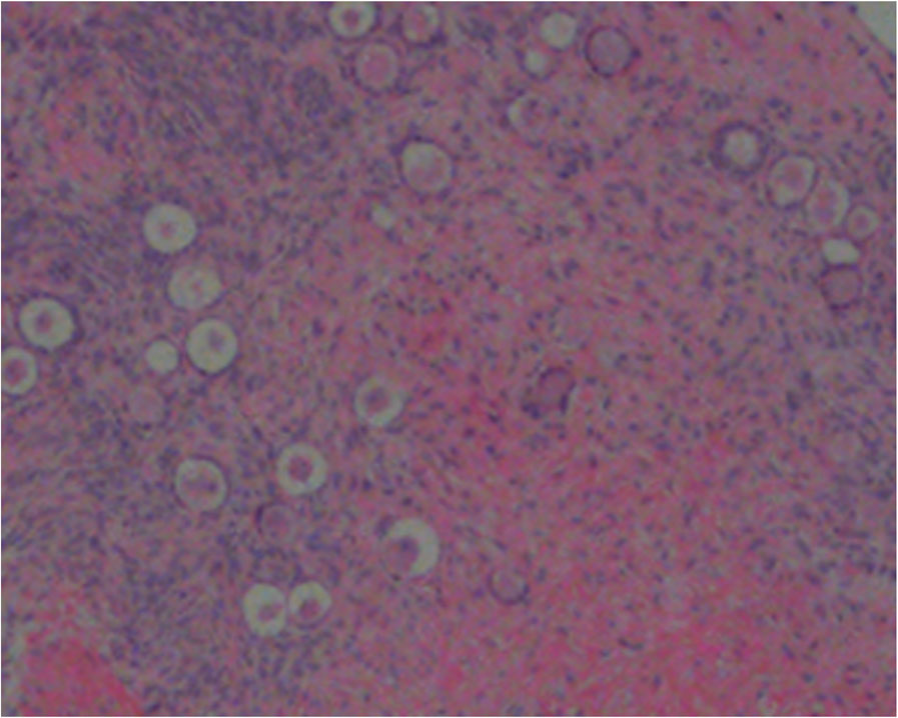
Primordial follicles from nude mice 2.5 months after human tissue transplantation into OBC stained with haematoxylin and eosin (4×). OBC: ovarian bursa cavity

**Fig. 3:**
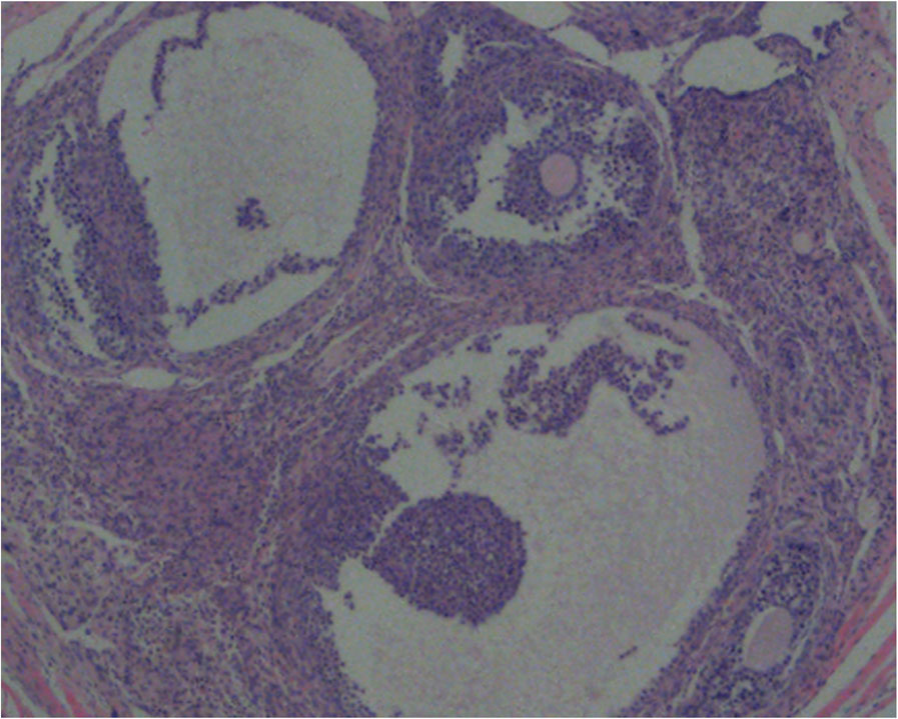
Antral follicles from nude mice 2.5 months after human tissue transplantation into OBC stained with haematoxylin and eosin (4×). OBC: ovarian bursa cavity

We counted the different stages of follicles to test whether the thawed ovarian tissues had survived or died. Soon after the ovarian tissue thawing, 150 primordial follicles were observed. No antral follicle was observed. Morphological analysis of 220 ovarian follicles from 12 nude mice were performed in one fragment (Fig. 2). Five antral follicles were observed in the ovarian bursa cavity group in one fragment (Fig. 3). TFSR of the ovarian bursa cavity group, the subcutaneous thigh group and the subcutaneous neck group were 100%.

The FGR in subcutaneous thigh transplantation group was 0%. The FGR in the ovarian bursa cavity transplantation group and subcutaneous neck transplantation group was not significantly different (*p* > 0.05) (Table 1).

**Table 1:** TFSR and FGR of frozen thawed human ovarian tissue after transplantation into nude mice

Groups	Primordial follicles	Primary follicles	Secondary follicles	Antral follicles	Albicans	TFSR(%)	FGR(%)
subcutaneous neck	62	3	0	0	5	100	4.62
ovarian bursa cavity	89	5	0	5	7	100	10.1
subutaneous thigh	56	0	0	0	3	100	0
*χ* ^2^	–	–	–	–	–	–	1.62
*p* -value	–	–	–	–	–	–	0.20*

Note: *Considering the FGR of subutaneous thigh is 0, *χ*^2^ test for FGR between subcutaneous neck group and ovarian bursa cavity group was only conducted, which showed there was no statistical difference between those two groups (*p* > 0.05);TFSR (total follicle surviving rate),FGR (follicle growth rate)

Frozen thawed human ovarian tissues were recovered 1.5 or 2.5 months after transplantation (Table 2). For both time periods the TFSR of two groups were 100%. FGR 2.5 months after transplantation was higher than FGR 1.5 months after transplantation (*p* < 0.05).

**Table 2:** TFSR and FGR of frozen thawed human ovarian tissue in two different periods after transplantation into nude mice

Groups	Primordial follicles	Primary follicles	Secondary follicles	Antral follicles	Atretic follicles	Albicans	TFSR (%)	FGR (%)
1.5 m after transplantation	104	2	0	0	0	5	100	1.9
2.5 m after transplantation	103	4	0	5	0	2	100	8.0
*χ* ^2^	–	–	–	–	–	–	–	4.30
*p* -value	–	–	–	–	–	–	–	0.04*

Note: *FGR in two different periods after transplantation into nude mice was significantly different (*p* < 0.05)

*TFSR* total follicle surviving rate

*FGR* follicle growth rate

Eleven of the twelve nude mice survived up to the end of the experiments. One died one week after transplantation. Thirty out of thirty-three ovarian grafts were recovered from the remaining eleven mice. The TRR was 90.91% (30/33). Ovarian tissue survival rate was 100% in the three groups. The TRR was 80% in ovarian bursa cavity group. The TRR was 100% in the subcutaneous neck transplantation group and subcutaneous thigh transplantation group. The TRR was not significantly different in the analyses between the three groups (*p* > 0.05) (Table 3).

**Table 3:** TRR of frozen thawed human ovarian tissue after transplantation into nude mice

Groups	Number of surviving nude mice	Total number of ovary tissues	Number of recovered tissues	TRR(%)	Survival rate(%)
subcutaneous neck	11	22	22	100	100
ovarian bursa cavity	11	20	16	80.0	100
subutaneous thigh	11	22	22	100	100
*χ* ^2^	–	–	–	2.82	–
*p* -value	–	–	–	0.09*	–

Note: *TRR was not significantly different in three groups (*p* > 0.05);TRR (tissue recovery rate)

### The serum FSH, E2 and AMH levels in nude mice

The level of FSH was not significantly different between the transplantation group and the control group (5.41 ± 1.73 mIU/ml vs 5.31 ± 1.46 mIU/ml) (*p* > 0.05), but both were significantly lower than in the ovariectomized group (9.12 ± 3.56 mIU/ml) (*p* < 0.05) (Table 4).

**Table 4:** FSH, AMH, E2 values comparison across three groups ($\overline{x}\pm s$)

Comparison Group	FSH(mIU/ml)	AMH(pg/ml)	E2(pg/ml)
Transplantation group	5.41 ± 1.73	342.71 ± 166.82	243.45 ± 234.47
Control group	9.12 ± 3.56	294.79 ± 152.42	254.69 ± 373.86
Ovariectomized group	5.41 ± 1.73	203.24 ± 32.47	64.54 ± 295.48
*F*	9.23	4.29	2.05
*p*-value	0.00	0.02	0.04

Note: After ANOVA test, the post-hoc analysis showed that there were statistical difference for FSH(Ovariectomized group VS Control group, Transplantation group VS Ovariectomized group, with *p*-value of 0.00 and 0.00, respectively), for AMH(Ovariectomized group VS Control group, Transplantation group VS Ovariectomized group, with *p*-value of 0.02 and 0.00, respectively),for E2(Ovariectomized group VS Control group, Transplantation group VS Ovariectomized group, with *p*-value of 0.04 and 0.04, respectively), whereas there were no statistical difference for FSH(Transplantation group VS Control group,with *p*-value of 0.86), for AMH(Transplantation group VS Control group,with *p*-value of 0.30),for E2(Transplantation group VS Control group,with *p*-value of 0.55).FSH: follicle stimulating hormone(mIU/ml);AMH:Anti-Mullerian hormone(pg/ml);E2:estradiol(pg/ml)

The level of E2 in transplantation group (243.45 ± 234.47 pg/ml) and control group (254.69 ± 373.86 pg/ml) was higher than the level in the ovariectomized group (164.54 ± 295.48 pg/ml) (*p* < 0.05). The level of E2 in transplantation group (243.45 ± 234.47 pg/ml) and control group (254.69 ± 373.86 pg/ml) was not significantly different (*p* > 0.05) (Table 4).

The level of AMH in transplantation group (342.71 ± 166.82 pg/ml) and control group (294.79 ± 152.42 pg/ml) was higher than the level in ovariectomized group (203.24 ± 32.47 pg/ml) (*p* < 0.05). The levels of AMH in transplantation group (342.71 ± 166.82 pg/ml) and control group (294.79 ± 152.42 pg/ml) were not significantly different (*p* > 0.05) (Table 4).

## Discussion

The quality of tissue only can be tested in vivo, therefore an animal model is needed. Testing the quality of tissue is decisive for the success of re-transplantation, and we consider it justifiable to use a mouse model from an ethical aspect. This also was confirmed by the university’s Ethics Committee. One main result of our study is that the endocrine function of the ovariectomized mice was restored after human ovarian tissue was transplanted. Another important result is that the ovarian fossa site was considered the best place for transplantation because antral follicles were only observed in the ovarian bursa cavity group, and FGR in the ovarian bursa cavity transplantation group was not significantly different compared to the neck transplantation group (*p* > 0.05). However, secondary follicles were not found in either group, maybe because we only observed three transplantation sites and two different periods after transplantation.

Transplantation sites are important for the survival and functional recovery of the ovaries. Several transplantation sites have been investigated for human ovarian tissue xenografting, including intraperitoneal, ovarian bursa cavity, kidney capsule, intramuscular and subcutaneous sites. However, the most appropriate site for an adequate clinical application has not yet been clearly determined [22–25].

Because the skin of nude mice is loose, providing enough space for the growth of grafts and ease of observation, we chose two sites for subcutaneous transplantation, the thigh and neck. In addition, we chose the ovarian bursa cavity because the transplantation is easy and quick to perform. According to our results, the ovarian bursa cavity was the best site. One explanation may be that the ovarian bursa cavity is rich in blood supply.

The FGR assessed at 2.5 months after transplantation was significantly higher than the FGR at 1.5 months (*p* < 0.05). The TFSR in both groups were 100%. Five antral follicles were seen after 2.5 months of transplantation. Other studies using xenografting models have also shown successful follicular development after long-term transplantation of human cryopreserved ovarian tissue to mice [15, 26]. One study showed that the proportion of frozen-thawed primordial follicles found after 28 weeks of grafting is comparable to that found after 3 weeks of grafting (36% vs. 41%) [15]. Likewise, in our experiments the cryopreserved ovarian cortex grafted into the nude mice was able to sustain ovarian tissue function. No albicans (white fibrous scar in an ovary produced by the involution of the corpus luteum) was found before cryopreservation and transplantation of the human ovarian tissue, and the appearance of albicans can also indicate that our cryopreservation technique was successful.

Our study showed that both the TRR of the subcutaneous neck and subcutaneous thigh sites were 100%. The TRR of ovarian bursa cavity was 80%. The graft survival rate was 100%. Kim et al. reported that the TRR of subcutaneous transplantation was 80% [22], which was lower than our experiment. Abir el al. [27] showed that the grafts survival rate was 50%, which was lower than in our study. Compared to these results, our cryopreservation technique was very successful.

In our study the levels of FSH, AMH and E2 were not significantly different between the control group and transplantation group (*p* > 0.05). We can therefore conclude that the ovarian tissue’s function in the transplanted group was similar to that of the control group. Transplantation of the human ovarian tissue could restore the endocrine function of the ovariectomized mice.

In conclusion, the studies which already have been performed to test the quality of tissue for ovarian tissue cryopreservation by xenograft transplantation have been controversial regarding the efficacy of this method and did not come to a conclusion about the best site for transplantation. However, our technique used in this randomized study compared with two controls (one with ovaries without any surgery, one ovariectomized group) was successful, and we have been able to identify the ovarian fossa site as the best site for transplantation. Thus, as demonstrated by testing for growing and developing follicles after xenotransplantation, the mouse model can be used to the quality of human ovarian tissue after cryopreservation. This is the precondition for successful re-transplantation after performing chemo- or/and radiotherapy in patients with certain cancer types, and is the technique used in our new centre for fertility protection, the first in China.

## Limitations

We only used cancer tissue from three patients to test the validity of the xenograft mouse model. However, we had a relatively large sample of mice (36) and strips (72) and each mouse received 6 strips for the 3 different sites to test whether the mouse model is appropriate to assess the quality of human cryopreserved tissue. In initiating the first “Fertility Protection Centre” using the cryopreservation technique in China, we certainly will be able to test more patients with different cancer types, and this will be part of our future research.
